# Personality and Perceived Health in Spousal Caregivers of Patients with Lung Cancer: The Roles of Neuroticism and Extraversion

**DOI:** 10.1155/2016/5659793

**Published:** 2016-04-07

**Authors:** Michael Hoerger, Maria Coletta, Silvia Sörensen, Benjamin P. Chapman, Kim Kaukeinen, Xin Tu, Paul R. Duberstein

**Affiliations:** ^1^Departments of Psychology, Psychiatry, and Medicine, Tulane Cancer Center, 3070 Stern Hall, New Orleans, LA 70118, USA; ^2^Main Line Health, Newtown Square, PA 19073, USA; ^3^Department of Psychiatry, University of Rochester Medical Center, 300 Crittenden Boulevard, Rochester, NY 14642, USA; ^4^Department of Biostatistics, University of Rochester Medical Center, 300 Crittenden Boulevard, Rochester, NY 14642, USA

## Abstract

*Purpose*. Family members' responsibilities for patients with cancer have increased dramatically over the past decade and will likely continue to rise. Given that caregiving is associated with declines in self-care, there is a need for research on caregivers' perceptions of their own health. The purpose of this study was to examine whether personality is associated with four self-report perceived health items from the SF-36.* Methods*. The sample consisted of 114 spouses of lung cancer patients who completed cross-sectional measures as part of a larger cohort study on adjustment to the diagnosis and treatment of lung cancer. Predictors of interest were Neuroticism and Extraversion scores from the NEO-FFI. Covariates were age, gender, conscientiousness, depressive symptoms, and objective illness burden.* Results*. Multivariate analyses revealed that caregivers with higher Extraversion scores were less likely to respond affirmatively to the item “I expect my health to get worse” (OR = 0.90, *p* < 0.05). Neuroticism was associated with poorer perceived health (ORs from 1.11 to 1.12, *p*'s < 0.05).* Conclusions*. The present cross-sectional findings suggest that personality is associated with responses to SF-36 perceived health items beyond what can be accounted for by objective illness burden and other covariates. The potential overestimation of health among extraverted caregivers may have implications for their health outcomes.

## 1. Introduction

Family members' caregiving responsibilities for patients with cancer have increased dramatically over the past decade and will likely continue to rise as care is now routinely administered on an outpatient basis and patients are living longer [1]. Understanding the psychological factors that contribute to morbidity among these “hidden patients” [2] may help improve the clinical assessment process and mitigate some of the adverse consequences of caregiving.

Perceived health is an important predictor of morbidity and mortality across various cultural contexts [3–7]. Subjective rating scales are often used to assess perceived health and are straightforward and quick to administer in busy clinical settings. Given the widespread use of these rating scales and their constituent items, it is important to identify influences on responses. In this study, we sought to examine whether personality is associated with perceived health in spouses of patients with lung cancer.

Although the term “caregiving” was initially used to refer to the provision of dementia care by family members, it is applicable to cancer care as well. Family members are not merely bystanders in treatment but also actual or potential cousers of services [8–10]. Family caregivers frequently accompany the patient to appointments, administer medications, and provide other critical day-to-day functions. For patients with lung cancer, where the 5-year survival rate is only 17% [11], spouses often provide in-home care. Recognizing that many caregivers derive psychological benefit from the provision of care [10, 12, 13], up to 30% of cancer caregivers experience significant psychological distress [10, 14–16]. Caregivers of patients diagnosed with lung cancer report more depressive symptoms than other caregivers [17].

Research also suggests that caregivers' physical health diminishes over time [18]. Meta-analyses have found that caregivers have poorer physical health [18, 19], which may result from self-neglect due to caregiving demands and the belief that responding to the health needs of one's spouse is more important than attending to one's own. In particular, caregivers may implicitly compare their health needs with those of their sick spouses [20], leading to a response shift [21, 22] in how they evaluate their own health. This shift might lead caregivers to care for their ill family members at the expense of their own health because they are “healthier” [20].

*Personality and Perceived Health*. Individual differences in how spouses respond to caregiving demands are now well documented [10, 20, 23, 24], and several studies have examined the personality correlates of perceived health [25–29]. To the best of our knowledge, only one study [2] has examined the personality correlates of health-related quality of life in the caregiving context, and that study did not examine specific perceived health items. Moreover, that study did not control for objective illness burden, so it was unclear whether personality was related to biased health perceptions or merely illness burden. In the current study, we controlled for objective illness burden [30] while examining caregivers' responses to four self-report perceived health items from the SF-36 [31]. These items were as follows: “I am as healthy as anybody I know,” “My health is excellent,” “I seem to get sick a little easier than other people,” and “I expect my health to get worse.”

Based on previous research from Chapman and colleagues [26], we derived two sets of hypotheses. The first concerns Neuroticism, a personality dimension involving negative affectivity and emotional instability, which has been associated with more health complaints in general samples [32, 33]. We hypothesized that caregiver Neuroticism would be associated with reports of poorer perceived health on all four items, above and beyond the effects of covariates. The second hypothesis concerns Extraversion, a personality dimension marked by positive affect and sociability. Individuals high in Extraversion tend to have positive views about the future [34, 35] and higher levels of emotional well-being, which may lead to more optimistic health assessments [33, 36]. People who are high in Extraversion may underestimate their symptoms and report overly positive health [37]. Consistent with findings from Chapman et al. [26] we hypothesized that Extraversion would be associated with the item tapping future expectations (“I expect my health to get worse”) but not with the other three items.

## 2. Methods

### 2.1. Participants and Procedures

The study was approved by the IRB at the University of Rochester. The sample consisted of spouses of patients with lung cancer who completed cross-sectional measures as part of a larger cohort study conducted in the Rochester, New York, region [14, 38]. As a nonintervention study, the parent investigation examined the natural course of adjustment to the diagnosis and treatment of lung cancer. Spouses of patients who had been diagnosed with and treated for lung cancer were eligible to participate.

A member of the research team identified eligible spouses, through introduction by the surgeon or oncologist. The research team member explained the nature of the study and invited the spouse to participate. Of the 340 spouses of lung cancer patients who were approached, 159 (47%) provided written consent for participation, and complete data were available for 114 (72% of those who consented). Informed consent was obtained at the time of the research interview; spouse participants also agreed to allow the research team to review their primary care medical chart. Researcher training sessions were conducted throughout the study to monitor rater drift and ensure the methodological integrity of the data collection process. Trained research assistants administered self-report questionnaires and observer-rated assessment instruments.

### 2.2. Measures

Personality was assessed with the NEO-Five Factor Inventory (NEO-FFI) [39]. From the NEO-FFI, we selected two specific indicators of personality: Neuroticism and Extraversion. Because it is consistently associated with both health behaviors and objective health indicators [40–42] we controlled for the personality dimension conscientiousness. Internal consistency in this study was good for Neuroticism (Cronbach's *α* = 0.84) and conscientiousness (*α* = 0.83), while being lower for Extraversion (*α* = 0.63). The NEO inventories have been used in prior research on spouses of chronically ill patients [23, 42, 43] and their validity has been well documented [39].

The Cumulative Illness Rating Scale (CIRS) [44] was used to ensure that any relationships between personality and subjective health were not confounded by objective illness burden. The CIRS is a validated [30, 45] physician-rated objective health index derived by means of patient history as well as physical examination and laboratory findings and quantifies the amount of physical disease in each organ system at the time of study entry. A physician-researcher reviewed the spouses' medical charts. Higher scores indicate greater disease burden.

Depressive symptoms were controlled for by using the 24-item observer-rated Hamilton Depression Rating Scale (HDRS) [46], which assesses the presence and severity of depressive symptoms over the past week. Symptoms measured on the HDRS include depressed mood, feelings of worthlessness, helplessness, hopelessness, loss of interest in pleasurable activities, fatigue, and somatic complaints. Higher scores reflect greater depressive symptoms. Scores were based on self-report and nonverbal presentation. In the present study, the HDRS had excellent internal consistency (Cronbach's *α* = 0.81).

To measure subjective health, we dichotomized four items from the General Health Perceptions Scale of the SF-36 [31]. Following Chapman and colleagues [26], we used two positively valenced items (“I am as healthy as anybody I know” and “My health is excellent”) and two negatively valenced questions (“I seem to get sick a little easier than other people” and “I expect my health to get worse”). Respondents who endorsed “neutral,” “definitely true,” or “mostly true” in response to the positive items and “definitely false” and “mostly false” to the negative items were categorized as reporting good perceived health. Respondents who endorsed “neutral,” “definitely true,” or “mostly true” in response to the negative items and “definitely false” and “mostly false” to the positive items were categorized as having poor perceived health. We dichotomized the item scores because binary scores have more practical utility for health care practitioners, but we also conducted sensitivity analyses on ordinal scores.

### 2.3. Data Analysis

For each of the outcome variables, we conducted a logistic regression model [47]. Predictors included Neuroticism and Extraversion. Given that perceived health is influenced by sociodemographic factors [48], objective health [33], and depressive symptoms [25, 26], the analyses controlled for age, gender, conscientiousness, CIRS, and HDRS. We also ran the regression models using ordinal logits for perceived health with the following ordered categories: good, neutral, and poor.

## 3. Results

### 3.1. Participant Characteristics

Descriptive statistics are summarized in Table 1. Caregivers had a mean age of 63 and were predominantly female (71%), Caucasian (97%), and living with patients (98%). Their mean level of education was 13 years, and the median income bracket was $25,000–34,999 (USD). Patients were distributed among cancer stages, and most of the patients (79%) had completed some form of treatment at the time of the spouse's participation, with surgery being the most common form of treatment, followed by combination therapy (surgery, radiation, and chemotherapy).

Table 2 shows the mean Neuroticism and Extraversion scores of those who endorsed good versus poor perceived health for each of the four SF-36 questions. The means and standard deviations for Neuroticism and Extraversion were comparable to those observed in large samples of healthy US adults [49].

### 3.2. Hypothesis Testing

Table 3 shows the significant predictors in the multiple regression analysis. Higher Neuroticism was significantly associated with poor perceived health measured by the item “I seem to get sick a little easier than other people” (*p* < 0.05). Higher Neuroticism was also a significant predictor for the endorsement of “I expect my health to get worse” (*p* < 0.05). Spouses who reported being more extraverted were less likely to endorse the item “I expect my health to get worse” (*p* < 0.05).

Among the control variables, the CIRS objective illness burden scores (*p* < 0.01) and age (*p* < 0.05) were associated negatively with “My health is excellent.” Older respondents were more likely to endorse “I expect my health to get worse” compared to younger respondents (*p* < 0.05). Conscientiousness and HDRS (depressive symptoms) were not significantly associated with responses to any of the perceived health items. Results did not significantly change when the analyses were conducted using ordinal logits.

## 4. Discussion

The purpose of this study was to test hypotheses about the relationship between two personality dimensions—Neuroticism and Extraversion—and responses to individual items tapping perceived health. Support for our hypotheses about Neuroticism was mixed. Neuroticism was found to be significantly associated with poor perceived health on the negatively worded items (“I seem to get sick a little easier than other people” and “I expect my health to get worse”), but not on the positively worded items (“My health is excellent” and “I am as healthy as anybody I know”). Findings for the negatively worded items are consistent with previous research [26, 32, 50]. Whereas Chapman and colleagues [26] reported significant associations between Neuroticism and the positively valenced items, there are at least four possible explanations for our failure to do so [51]. First, responses to the positively valenced items “My health is excellent” and “I am as healthy as anybody I know” may implicitly require caregiver respondents to make a judgment about their health in comparison to their ill spouse. A second and related point is that very few people reported poor perceived health on the item “I am as healthy as anybody I know,” reducing statistical power. Third, the Chapman et al. sample was older, and some of Neuroticism's effects on perceived health may not become observable until later adulthood [25, 52]. Finally, Neuroticism may confer a greater sensitivity to the presence of negative affect and negatively valenced items as opposed to the absence of positive affect [53]. This hypothesis could be examined in future clinical research. If it is supported, it would underscore for clinicians and researchers the importance of language, word choice, and item framing [54].

As hypothesized, people with higher Extraversion scores are less likely to endorse the item “I expect my health to get worse,” controlling for physician assessment of actual health status based on medical records. Extraversion may be particularly relevant in the caregiving context because extraverted people tend to be optimistic and feel comfortable in the presence of others. People who are higher in Extraversion tend to have a more positive outlook on life. A factor termed “optimistic control,” characterized by optimistic expectation for life outcomes, positive self-esteem, hope, and internal control, is positively correlated with Extraversion [34]. Additionally, positive affect has been shown to underlie thoughts about the future more than negative affect [53, 55, 56], which may impact how individuals higher in Extraversion judge the possibility of future health declines.

Assuming that middle-aged and older adults can expect their health to deteriorate over time and that caregivers are at increased risk for such deterioration, caregivers lower in Extraversion may make more accurate judgments about their health. In contrast, those higher in Extraversion may overlook important signs and symptoms of disease and fail to report these to a physician. Although a positive outlook on life has many physical and mental health benefits [57], some studies suggest that unrealistic optimism about the future in the face of vulnerability to health issues may undermine specific risk reduction behaviors [58, 59]. Extraverted individuals may be identifiable in the consulting room [60]. For example, extraverts often dominate conversations, speak loudly, are gregarious, and are physically animated and enthusiastic [61]. Health care providers are advised to attend to more subtle cues for physical illness among extraverted caregivers and regard with empathy and skeptical curiosity the extravert's appraisals of their own health.

Patient-reported outcomes (PROs) are increasingly being integrated into clinical research, care, accreditation standards, and reimbursement rates [62–65], so studies evaluating the psychometrics of commonly used measures, such as the SF scales, are timely. We found, unsurprisingly, that control variables of objective illness burden and age were associated with perceived health, which underscores the importance of controlling for these variables in research aiming to understand biased health perceptions. One prior study has linked personality to perceived health in a caregiver sample [2], and ours is the first to do so while controlling for objective illness burden. If personality can shape responses to self-reported health items, independent of objective illness burden, clearly more research is needed aimed at understanding psychosocial factors that may bias scores on self-reports of health status and other PROs, such as patient satisfaction with care.

Several study strengths and weaknesses should be noted. This is the first study of which we are aware to examine personality and perceived health in a caregiving context while controlling for objective illness burden. Additional strengths include rigorous controls for other potential confounders like depression and careful analysis of specific SF-36 questions, a level of detail seldom pursued, despite the fact that the wording of the questions varies greatly and that clinical interviews typically assess health through a few such individual questions, rather than a formal composite score. The main limitation is the correlational design. Causal inferences cannot be drawn. Also, the internal consistency reliability of the Extraversion measure was lower than desirable, so the observed effects may have underestimated the association between Extraversion and perceived health. Finally, although levels of Neuroticism and Extraversion in this sample were comparable to those reported in national samples [49] and we have personality data on more than 70 percent of the cohort, generalizability to the entire cohort of consenting participants cannot be guaranteed.

The goal of the present investigation was to examine the association between personality and perceived health in caregivers of patients with lung cancer. An important next step would be to extend these findings by examining moderators of the association, such as demographic and health characteristics. For example, we did not examine health behaviors, such as smoking, drinking, and drug use, and it is possible that the association between Extraversion and optimistic reports of health is stronger among individuals who avoid these behaviors and consequently experience fewer daily reminders of ill health (e.g., coughing and hangovers). Additionally, the association between Neuroticism and poorer perceived health could be stronger for caregivers who have fewer economic resources, whose care receiver has a worse prognosis, or who spend more time providing care.

In closing, the present findings suggest that personality is associated with how spouses of cancer patients think about their health. Given that perceived health has prognostic implications for a variety of health outcomes and the mounting evidence for the role of personality in health and longevity [66, 67], it is important to explore ways to assess personality in clinical settings in order to target and tailor efforts to modify potentially inflated or deflated misperceptions of one's own health. Understanding the factors that contribute to perceived health threats among caregivers can help prevent the commonly observed negative effects of caregiving. This information is of value to the health care providers who care for cancer patients or their spouses.

## Figures and Tables

**Table 1:** Descriptive statistics.

Variable	M (SD) or *N* (%)
Age, years	63.4 (9.9)
Gender, female	81 (71.1%)
Education, years	13.0 (2.1)
Income	
<$10,000	2 (1.8%)
$10,000–24,999	24 (21.1%)
$25,000–34,999	31 (27.2%)
$35,000–49,999	31 (27.2%)
$50,000 or higher	26 (22.8%)
Race, Caucasian	111 (97.3%)
Residing with patient	112 (98.2%)
Cancer stage	
I	54 (47.4%)
II	14 (12.3%)
III	28 (24.6%)
IV	18 (15.8%)
Cancer treatments	
Surgery only	50 (43.9%)
Radiation only	5 (4.4%)
Chemotherapy only	3 (2.6%)
Combination therapy	32 (28.1%)
No treatments	24 (21.1%)

*Note*. *N* = 114. Percentages may not sum to 100.0% due to rounding.

**Table 2:** Personality dimensions associated with good and poor perceived health.

SF-36 item	Perceived health	Neuroticism M (SD)	Extraversion M (SD)
“I seem to get sick a little easier than other people”	Good (*n* = 107)	16.8 (5.39)	28.1 (5.34)
	Poor (*n* = 5)	20.0 (5.57)	27.2 (6.14)

“I am as healthy as anybody I know”	Good (*n* = 88)	16.9 (5.23)	28.4 (4.73)
	Poor (*n* = 24)	16.9 (6.19)	27.1 (7.23)

“I expect my health to get worse”	Good (*n* = 99)	17.1 (5.37)	28.3 (5.44)
	Poor (*n* = 13)	15.6 (5.80)	26.2 (4.36)

“My health is excellent”	Good (*n* = 89)	16.3 (5.14)	28.7 (4.76)
	Poor (*n* = 23)	19.1 (6.00)	25.9 (6.86)

*Note*. Perceived health responses were dichotomized.

**Table 3:** Personality dimensions and covariates associated with poor perceived health.

SF-36 item	Significant predictor	OR (95% CI)	*p*
“I seem to get sick a little easier than other people”	Neuroticism	1.11 (1.00–1.23)	0.05

“I am as healthy as anybody I know”	—		

“I expect my health to get worse”	Age	1.06 (1.01–1.13)	0.04
	Neuroticism	1.12 (1.02–1.23)	0.02
	Extraversion	0.90 (0.80–0.99)	0.05

“My health is excellent”	Age	0.94 (0.88–0.99)	0.05
	CIRS	1.51 (1.17–2.02)	0.003

*Note*. Categorical regression models are reported. Regression model using ordinal logits did not significantly change results. Covariates were age, gender, conscientiousness, Cumulative Illness Rating Scale (CIRS), and Hamilton Depression Rating Scale (HDRS).
